# Gradient boosting models for gallstone disease classification: A comparative study using demographic and clinical data

**DOI:** 10.1371/journal.pone.0361572

**Published:** 2026-10-30

**Authors:** Kazi Sakib Hasan, Sumaiya Hossain Puspita

**Affiliations:** 1 School of Data and Sciences, BRAC University, Dhaka, Bangladesh; 2 Department of R&D, Chowa Giken Corporation, Sapporo, Japan; 3 Department of Computer Science and Engineering, East Delta University, Chattogram, Bangladesh; National Taiwan University Hospital, TAIWAN

## Abstract

Gallstone disease is a prevalent gastrointestinal condition with a substantial healthcare burden. While abdominal ultrasonography is the established first-line diagnostic modality, opportunistic risk stratification using routinely collected health parameters remains underexplored. This study evaluated the discriminatory capacity of four gradient boosting machine learning algorithms (XGBoost, LightGBM, CatBoost, and HistGBM) for classifying gallstone status using demographic, bioimpedance, and routine laboratory features. A retrospective clinical dataset of 319 patients (161 gallstone-positive individuals and 158 healthy controls) from Ankara VM Medical Park Hospital was analyzed. Model performance was assessed using repeated stratified 10-fold cross-validation, evaluated via AUC-ROC, accuracy, F1-score, sensitivity, specificity, Brier score, and decision curve analysis (DCA), with SHAP analyses employed for model interpretability. CatBoost achieved the highest discriminatory performance (AUC = 0.862±0.061, accuracy = 0.794±0.075) and the lowest Brier score (0.149±0.037), demonstrating statistically significant superiority over competing architectures (Friedman test, *p* < 0.001). Feature ablation revealed that bioimpedance parameters alone provided substantial independent discriminative signal (AUC = 0.797±0.079), approaching that of laboratory markers (AUC = 0.829±0.070), whereas demographic attributes alone were insufficient (AUC = 0.476±0.097). SHAP analyses identified C-reactive protein, Vitamin D, and bone mass as the most influential variables, alongside non-linear risk associations for visceral fat area and body mass index. Decision curve analysis demonstrated positive net clinical benefit across threshold probabilities of 0.10–0.80. These findings demonstrate that gradient boosting models, particularly CatBoost, can provide robust, interpretable retrospective classification of gallstone disease, offering proof-of-concept potential for opportunistic risk stratification in metabolic care settings and pre-imaging triage for ambiguous outpatient presentations rather than replacing first-line ultrasonography.

## 1. Introduction

Gallstone disease, or cholelithiasis, represents one of the most prevalent and costly gastroenterological disorders globally, imposing a substantial burden on healthcare systems. The pathogenesis of gallstones is complex and multifactorial, closely linked to metabolic syndrome, aging, and dietary habits [1]. While many patients remain asymptomatic, a significant proportion develop severe complications, including acute cholecystitis, biliary colic, and acute pancreatitis, which often necessitate surgical interventions such as cholecystectomy [2]. The escalating incidence of gallstone disease, coupled with the high economic costs associated with surgical management and hospitalizations, underscores the critical need for early, non-invasive risk stratification and objective clinical decision-support strategies [3].

In recent years, the integration of Artificial Intelligence (AI) and Machine Learning (ML) has revolutionized the diagnostic landscape of hepatobiliary diseases. A substantial portion of applied AI research in this domain has historically focused on computer vision techniques applied to medical imaging, such as automated gallstone detection via computed tomography (CT) [4]. However, while imaging via AI is definitive for diagnosis, developing such models is computationally costly. Consequently, predictive modeling utilizing structured tabular data, including electronic health records (EHR), demographic profiles, bioimpedance measurements, and routine laboratory tests has attracted growing interest as a cost-effective, complementary strategy for non-invasive risk assessment and pre-test likelihood estimation [5]. These tabular ML approaches aim to leverage routinely collected clinical data to assist clinicians in identifying high-risk patterns dynamically [6].

Despite the high predictive accuracy of advanced ML algorithms, their clinical adoption is frequently hindered by their “black-box” nature. In healthcare, where automated predictions directly impact patient management, interpretability is as crucial as accuracy [7]. Explainable AI (XAI) frameworks have become indispensable in clinical modeling to establish clinician trust and ensure that model outputs align with medical pathophysiology. Techniques such as SHapley Additive exPlanations (SHAP) have been increasingly applied to clinical risk prediction models, providing transparent, patient-level insights into how individual risk factors, such as lipid profiles or systemic inflammation, contribute to biliary disease development [8,9].

Among the diverse array of ML architectures, Gradient Boosting Machines (GBMs) have inherent explainability and have also demonstrated exceptional efficacy in predictive healthcare modeling [10]. Recent studies also have successfully deployed gradient boosting variants to predict gallstone formation, choledocholithiasis, and post-operative complications with high sensitivity and specificity [11–13]. The success of these models is intrinsically linked to their algorithmic architecture. Extensive empirical research has consistently proven that tree-based gradient boosting models, such as XGBoost, LightGBM, and CatBoost, maintain a distinct dominance over deep learning algorithms when applied to medium-sized, structured tabular datasets [14]. They are inherently resilient to heterogeneous data types, robust against outliers, and capable of capturing complex, non-linear clinical interactions without requiring exhaustive data preprocessing or scaling [15,16].

To the best of our knowledge, no prior study has carefully evaluated gradient boosting models on a multi-domain gallstone dataset integrating demographic, bioimpedance, and laboratory variables, despite the strong empirical performance of gradient boosting methods on structured tabular datasets. This gap presents a unique opportunity to evaluate state-of-the-art tabular algorithms on a recent gallstone detection problem.

Therefore, this study aims to robustly evaluate and interpret multiple gradient boosting models for non-invasive gallstone disease classification and risk stratification. Specifically, our research is guided by the following primary research questions (RQs):

- **RQ1 — Discrimination Performance:** Can gradient boosting models accurately discriminate between gallstone-positive and gallstone-negative individuals using demographic, bioimpedance, and laboratory features?
- **RQ2 — Calibration Reliability:** How well-calibrated are the predicted probabilities generated by the evaluated machine learning models?
- **RQ3 — Clinical Utility and Interpretability:** Do the developed models provide clinically interpretable and potentially useful decision-support signals through SHAP-based explanations and Decision Curve Analysis (DCA)?

### 1.1. Literature review

Gallstone disease and related gallbladder disorders have been extensively studied using both imaging-based deep learning and structured-data machine learning approaches due to their high global prevalence and clinical burden. Recent advances demonstrate strong potential for artificial intelligence in improving diagnostic accuracy, risk stratification, and clinical decision support across multiple gallbladder-related conditions.

#### 1.1.1. Computer vision for gallstone and gallbladder disease detection.

Deep learning methods applied to medical imaging have shown high performance in gallstone detection and gallbladder disease classification. Meyer et al. [17] developed a CT-based deep learning system for automated gallstone detection, achieving 96.7% sensitivity and 83.3% specificity, with particularly strong performance on large and hyperdense stones. In ultrasound imaging, Mitu et al. [18] proposed an ensemble of CNNs and Vision Transformers for multi-class gallbladder disease classification, achieving 98% accuracy and 98.8% ROC-AUC across clinically relevant categories.

Further improvements in explainability and robustness have been reported through hybrid deep learning approaches. Kumar et al. [19] introduced an explainable CNN framework incorporating GAN-based augmentation and hybrid visualization methods, outperforming pretrained CNN and Vision Transformer models. Hasan et al. [20] proposed an anatomy-aware transformer model (GBCHV-Trans) achieving 96.21% accuracy for gallbladder classification. Similarly, Nabil et al. [21] developed MSFE-GallNet-X, a multi-scale CNN with Grad-CAM and LIME explainability, achieving 99.63% accuracy while maintaining computational efficiency.

#### 1.1.2. ML for gallstone and gallbladder disease prediction.

Beyond imaging, structured clinical data has been widely used for gallstone-related prediction tasks. Cetin et al. [22] applied multiple machine learning models to identify malignancy risk factors in gallstone disease, with TabPFN achieving the best predictive performance. Zeqo et al. [23] combined metaheuristic optimization with machine learning models, where Ant Colony Optimization with neural networks achieved 86.1% accuracy.

Wang et al. [24] developed an SVM + Random Forest hybrid model for differentiating gallbladder adenomas from cholesterol polyps, achieving high AUC values of 0.972 and 0.922 across validation cohorts. Ma et al. [25] introduced a CT radiomics-based machine learning model for predicting acute gallstone pancreatitis severity, achieving an AUC of 0.914 and demonstrating clinical applicability through an online decision tool.

#### 1.1.3. Interpretable AI in gallstone research.

Interpretability has become increasingly important in clinical machine learning applications. Rodriguez et al. [26] demonstrated that an SVM model with SHAP and LIME explanations achieved 88.8% AUC in gallstone risk prediction using bioimpedance and laboratory markers. Srivastava et al. [27] further extended interpretability to gallbladder cancer survival prediction using a stacking ensemble model with near-perfect performance (AUROC = 0.9949) supported by SHAP, PDP, ICE, and LIME explanations.

Other interpretable frameworks include Shrestha et al. [28], who proposed a rotational forest optimized with Bald Eagle Search achieving 78% accuracy, and Sarker et al. [29], who used Sand Cat Swarm Optimization with Random Forest achieving 81.25% accuracy while identifying key clinical predictors using SHAP and DiCE. Elbedwehy et al. [30] further demonstrated a transformer-based explainable framework achieving 99.86% accuracy with integrated XAI modules.

#### 1.1.4. Gradient boosting and ensemble learning for gallstone prediction.

Ensemble learning approaches, particularly stacking and boosting methods, have shown strong performance across gallstone-related prediction tasks. Steinway et al. [31] developed a gradient boosting model for choledocholithiasis prediction, outperforming clinical guidelines with an AUC of 0.79 and improving ERCP decision-making accuracy. Kanwal et al. [32] proposed a stacked ensemble model integrating multiple heterogeneous classifiers, achieving 97.0% accuracy with robust cross-validation performance.

Parthasarathy et al. [33] introduced GallIntel, a genetically optimized stacking ensemble combining Logistic Regression, XGBoost, AdaBoost, and Random Forest, achieving 90.62% accuracy with strong generalization performance.

#### 1.1.5. Research gap and motivation.

Despite the strong empirical performance of machine learning models in gallstone disease prediction, existing studies remain fragmented across imaging-based deep learning, tabular clinical modeling, and interpretability-driven approaches. In particular, ensemble learning methods such as gradient boosting have consistently demonstrated strong performance on structured datasets; however, their application has not been systematically evaluated on recently developed integrative gallstone datasets integrating demographic, bioimpedance, and laboratory variables.

To the best of our knowledge, no prior study has comprehensively investigated gradient boosting models under a unified experimental framework on such multi-domain clinical data. This gap highlights the need for a systematic evaluation of gradient boosting approaches to better understand their predictive capacity and clinical utility in gallstone disease diagnosis and risk stratification.

## 2. Materials and methods

### 2.1. Study design and data acquisition

This study was conducted using a retrospective clinical dataset obtained from the Internal Medicine Outpatient Clinic of Ankara VM Medical Park Hospital. The dataset comprises records from 319 patients collected between June 2022 and June 2023. The cohort is highly balanced, consisting of 161 individuals diagnosed with gallstone disease and 158 healthy controls. The study protocol received ethical approval from the Ankara City Hospital Ethics Committee (Approval Number: E2-23–4632). Following ethical guidelines, the data was completely anonymized before analysis. The dataset was subsequently deposited into the UCI Machine Learning Repository to facilitate reproducible research. The dataset is uploaded as a clean instance, which eradicates the need for further preprocessing [34]. The coding file of this research is available in a GitHub repository [35]. All statistical modeling and machine learning workflows were executed in Python (version 3.12.13), utilizing the scikit-learn (version 1.6.1), scipy (version 1.16.3), and shap (version 0.51.0) libraries.

The dataset encapsulates a multi-domain clinical profile consisting of 38 distinct features, which are categorized into three primary domains: demographic characteristics, bioimpedance measurements, and laboratory parameters. Baseline patient characteristics, as summarized in Table 1, were statistically compared between the gallstone-positive and gallstone-negative cohorts. Continuous variables are presented as mean ± standard deviation and were evaluated using independent samples t-tests (with Welch’s correction for unequal variances). Categorical variables are expressed as counts and percentages, analyzed via Pearson’s Chi-square ($\chi^{2}$) tests.

**Table 1:** Baseline dataset characteristics by gallstone status.

Feature	Gallstone (+)	Gallstone (-)	*p*-value
**Demographics**			
Age	48.51 ± 11.20	47.63 ± 12.97	0.5173
Gender	90 (57.0%)	67 (41.6%)	0.0086
Comorbidity	0.31 ± 0.46	0.36 ± 0.57	0.3869
Coronary Artery Disease (CAD)	3 (1.9%)	9 (5.6%)	0.1504
Hypothyroidism	3 (1.9%)	6 (3.7%)	0.5172
Hyperlipidemia	8 (5.1%)	0 (0.0%)	0.0113
Diabetes Mellitus (DM)	27 (17.1%)	16 (9.9%)	0.0880
Height	166.06 ± 10.25	168.23 ± 9.77	0.0543
Weight	81.34 ± 15.44	79.81 ± 15.98	0.3861
Body Mass Index (BMI)	29.53 ± 5.27	28.24 ± 5.29	0.0300
**Bioimpedance**			
Total Body Water (TBW)	39.70 ± 7.97	41.46 ± 7.81	0.0472
Extracellular Water (ECW)	16.50 ± 3.28	17.63 ± 2.94	0.0014
Intracellular Water (ICW)	23.44 ± 5.60	23.82 ± 5.10	0.5305
Extracellular Fluid/Total Body Water (ECF/TBW)	41.66 ± 3.58	42.76 ± 2.78	0.0024
Total Body Fat Ratio (TBFR) (%)	30.19 ± 8.06	26.39 ± 8.41	< 0.0001
Lean Mass (LM) (%)	69.72 ± 8.08	73.52 ± 8.38	< 0.0001
Body Protein Content (Protein) (%)	15.74 ± 2.54	16.14 ± 2.10	0.1242
Visceral Fat Rating (VFR)	9.16 ± 4.05	9.00 ± 4.61	0.7446
Bone Mass (BM)	2.69 ± 0.50	2.91 ± 0.50	< 0.0001
Muscle Mass (MM)	53.28 ± 11.00	55.25 ± 10.14	0.0965
Obesity (%)	41.82 ± 154.51	29.99 ± 21.60	0.3421
Total Fat Content (TFC)	25.14 ± 9.31	21.87 ± 9.65	0.0023
Visceral Fat Area (VFA)	12.92 ± 5.10	11.44 ± 5.33	0.0120
Visceral Muscle Area (VMA) (kg)	30.08 ± 4.09	30.73 ± 4.78	0.1929
Hepatic Fat Accumulation (HFA)	1.25 ± 1.07	1.06 ± 1.04	0.1077
**Laboratory Data (Labs)**			
Glucose	108.17 ± 40.57	109.20 ± 48.81	0.8376
Total Cholesterol (TC)	204.15 ± 48.28	202.86 ± 43.28	0.8021
Low Density Lipoprotein (LDL)	124.46 ± 40.93	128.80 ± 36.04	0.3153
High Density Lipoprotein (HDL)	52.31 ± 21.75	46.70 ± 12.00	0.0048
Triglyceride	139.49 ± 90.36	149.42 ± 104.83	0.3648
Aspartate Aminotransferase (AST)	19.41 ± 15.95	23.91 ± 17.16	0.0158
Alanin Aminotransferaz (ALT)	25.68 ± 31.48	28.01 ± 23.88	0.4566
Alkaline Phosphatase (ALP)	75.79 ± 22.99	70.48 ± 25.09	0.0497
Creatinine	0.78 ± 0.17	0.82 ± 0.18	0.0181
Glomerular Filtration Rate (GFR)	100.34 ± 17.77	101.29 ± 16.20	0.6152
C-Reactive Protein (CRP)	3.27 ± 6.34	0.46 ± 2.48	< 0.0001
Hemoglobin (HGB)	14.07 ± 1.75	14.76 ± 1.74	0.0004
Vitamin D	17.83 ± 9.58	24.90 ± 9.12	< 0.0001

The statistical analysis revealed several significant clinical distinctions between the two groups. Notably, individuals with gallstones exhibited higher overall adiposity and altered body composition metrics, including a significantly elevated Body Mass Index (*p* = 0.0300), Total Body Fat Ratio (*p* < 0.0001), and Visceral Fat Area (*p* = 0.0120). Furthermore, laboratory profiles indicated heightened systemic inflammation and metabolic variations in the gallstone-positive cohort, characterized most prominently by elevated C-Reactive Protein (*p* < 0.0001) and markedly reduced Vitamin D levels (*p* < 0.0001). Significant demographic variations were also observed, particularly concerning gender distribution (*p* = 0.0086) and a higher prevalence of hyperlipidemia (*p* = 0.0113) among patients with gallstone disease. Fig 1 shows the workflow of the study.

**Fig 1:**
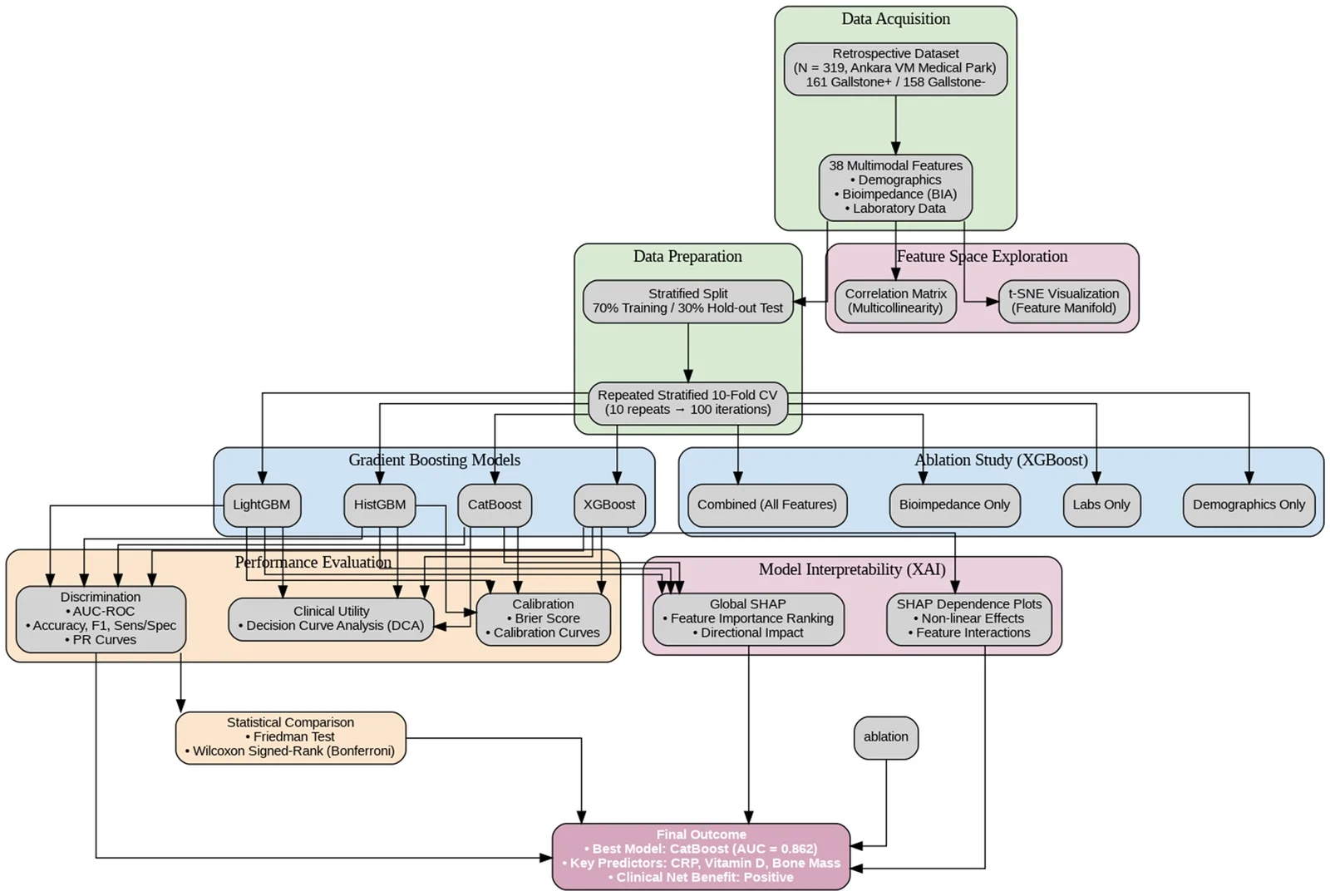
Study workflow. From data acquisition to model evaluation and interpretation (XGBoost, LightGBM, CatBoost, HistGBM).

### 2.2. ML Algorithms

To address the primary research question regarding discrimination performance (RQ1), four state-of-the-art gradient boosting frameworks were employed. These models are highly effective for tabular healthcare data due to their ability to capture complex, non-linear interactions without extensive scaling:

- **XGBoost (eXtreme Gradient Boosting):** Utilized for its efficient handling of sparse data and advanced regularization to prevent overfitting [36].
- **LightGBM (Light Gradient Boosting Machine):** Employs gradient-based one-side sampling and an exclusive feature bundling technique, optimizing both training speed and memory consumption [37].
- **CatBoost (Categorical Boosting):** Implements ordered boosting and oblivious trees, reducing prediction shift and intrinsically managing potential categorical permutations [38].
- **HistGBM (Histogram-based Gradient Boosting):** An implementation inspired by LightGBM, utilizing histogram-based binning of continuous features to dramatically accelerate the decision tree construction process [39].

All models were initialized with a fixed random seed (random_state = 42) to ensure strict computational reproducibility.

### 2.3. Training strategy and validation scheme

To robustly evaluate model generalizability and mitigate selection bias, a 10-fold Repeated Stratified Cross-Validation (RSCV) with 10 repetitions was implemented, yielding 100 independent training and testing iterations per model. For secondary analyses requiring static plotting (i.e., Probability Calibration, DCA, and SHAP visualizations), the dataset was partitioned into a 70% training set and a 30% hold-out testing set using stratified sampling to preserve the class prevalence.

Furthermore, an ablation study was constructed to isolate the predictive power of distinct feature sets. The primary XGBoost model was systematically trained and evaluated using exclusive subsets (Demographics only, Labs only, Bioimpedance only) and compared against the combined multi-domain dataset.

### 2.4. Performance evaluation metrics

Model efficacy was comprehensively evaluated across discrimination, calibration, and clinical utility:

- **Discrimination (RQ1):** Classification performance was quantified using Area Under the Receiver Operating Characteristic Curve (AUC-ROC), Accuracy, F1-Score, Sensitivity (Recall), and Specificity. Precision-Recall (PR) curves were generated to further validate performance.
- **Calibration Reliability (RQ2):** The alignment between predicted probabilities and actual outcomes was assessed using the Brier Score and visualized via 10-bin calibration curves.
- **Clinical Utility (RQ3):** DCA was employed to calculate the net clinical benefit of the models across a continuum of threshold probabilities, comparing the models against “treat-all” and “treat-none” default strategies.

### 2.5. Feature space visualization and correlation

To explore the underlying structure of the clinical dataset, a two-dimensional t-distributed Stochastic Neighbor Embedding (t-SNE) was generated (Fig 2). The visualization displays a largely overlapping manifold between patients with and without gallstone disease. This lack of clear linear separability suggests that non-linear algorithms, such as gradient boosting machines, are well-suited for this classification task. Furthermore, the correlation matrix (Fig 3) highlights the expected multicollinearity within bioimpedance compartments (e.g., Total Body Water and Extracellular Water) and reveals weak-to-moderate correlations between localized adiposity metrics and metabolic laboratory markers.

**Fig 2:**
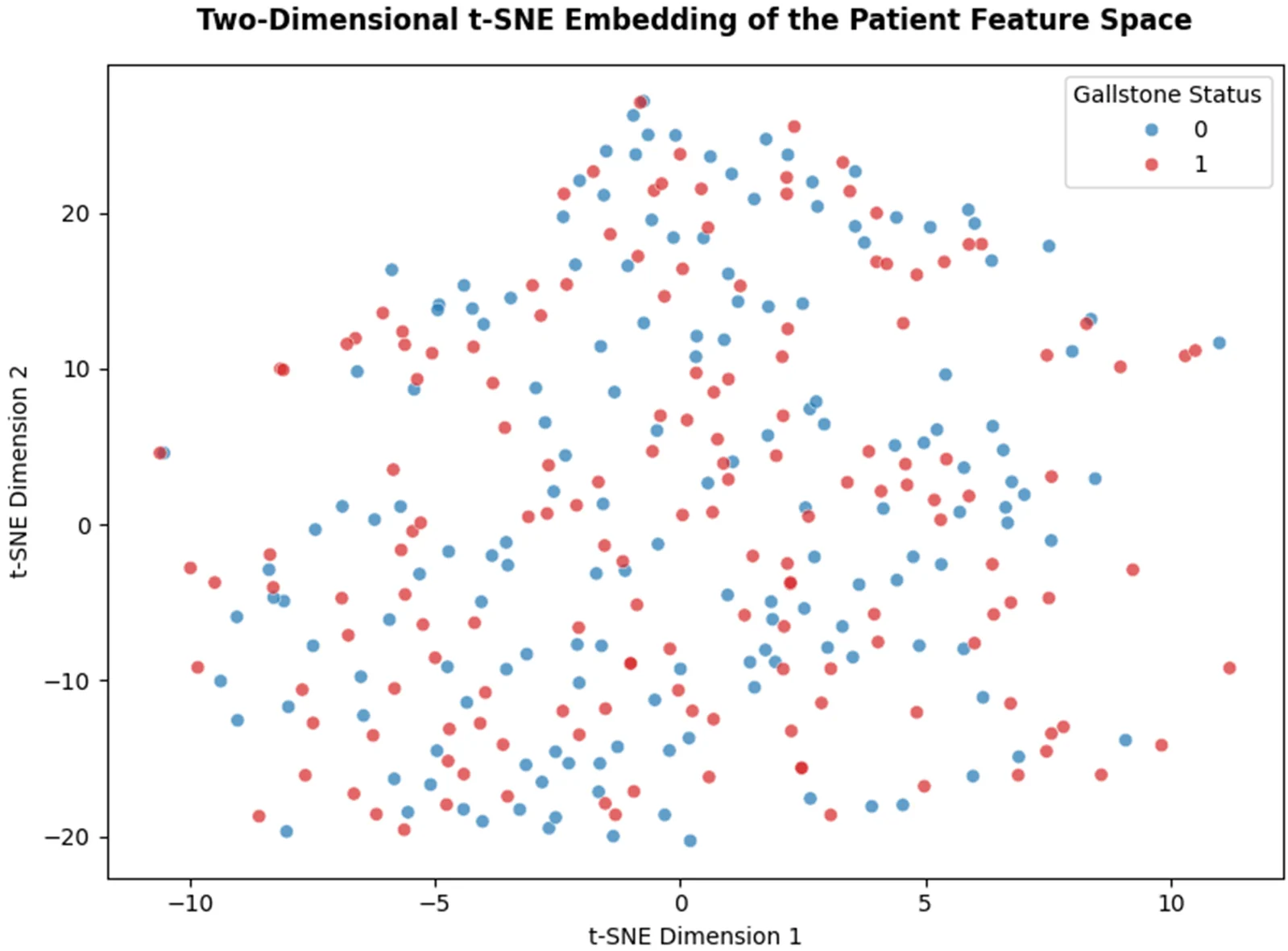
Two-Dimensional t-SNE Embedding of the Patient Feature Space. Red dots indicate positive gallstone status, while blue dots indicate negative status, demonstrating overlapping feature distributions.

**Fig 3:**
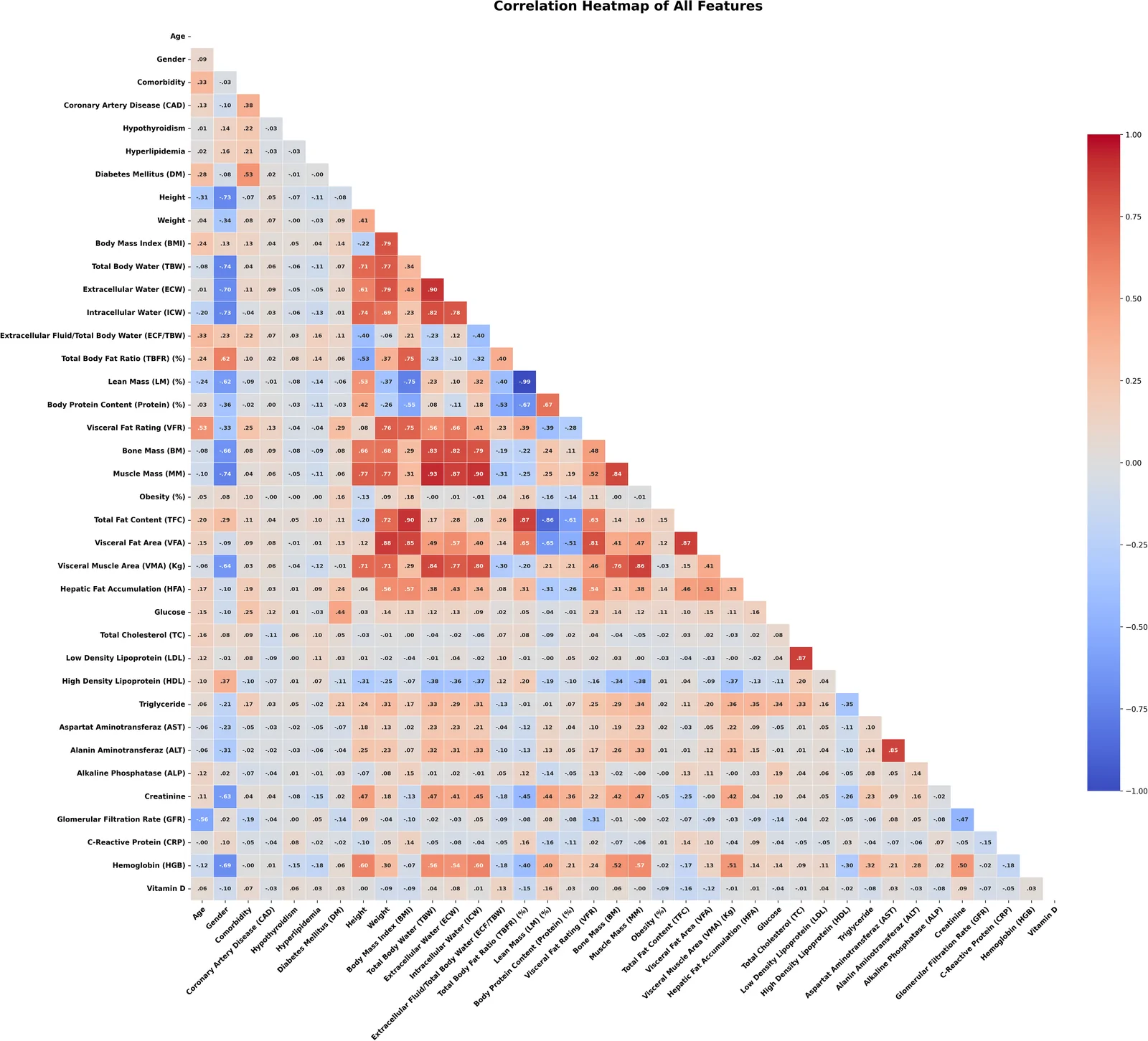
Correlation Matrix of Clinical, Laboratory, and Bioimpedance Variables. The heatmap explores multicollinearity, displaying strong intra-domain relationships among bioimpedance features and moderate cross-domain relationships.

### 2.6. Model interpretability

SHAP were utilized to explicate the “black-box” nature of the boosting models. Global SHAP summary plots were constructed for all four algorithms to rank feature importance and depict the directional impact of feature values on gallstone risk. To analyze specific non-linear effects and feature interactions, SHAP dependence plots were generated using the XGBoost model for six highly influential clinical features identified by domain literature: Triglyceride, HDL, ALT, Visceral Fat Area, Hepatic Fat Accumulation, and BMI.

### 2.7. Statistical analysis

A non-parametric omnibus Friedman test was conducted to rigorously compare the predictive performance (AUC) of the machine learning algorithms across the 100 cross-validation folds. Following a significant Friedman result, post-hoc pairwise comparisons were executed utilizing the Wilcoxon signed-rank test. To control the family-wise error rate across multiple comparisons, *p*-values were adjusted using the Bonferroni correction method. A two-tailed *p*-value < 0.05 was considered statistically significant for all tests.

## 3. Results

### 3.1. Model performance and calibration

As mentioned before, four gradient boosting architectures were evaluated using repeated stratified cross-validation. Table 2 shows that all models achieved comparable and robust predictive performance. CatBoost demonstrated a slightly higher discriminatory ability with an AUC of 0.862±0.061 and an accuracy of 0.794±0.075. It also exhibited the lowest (best) Brier Score (0.149±0.037), suggesting favorable probability calibration.

**Table 2:** Predictive Performance and Calibration Metrics of Evaluated Models.

Model	Accuracy	AUC	F1 Score	Sensitivity	Specificity	Brier Score
XGBoost	0.782 ± 0.068	0.847 ± 0.064	0.776 ± 0.075	0.770 ± 0.106	0.794 ± 0.100	0.171 ± 0.051
LightGBM	0.780 ± 0.066	0.845 ± 0.063	0.774 ± 0.074	0.771 ± 0.106	0.788 ± 0.095	0.168 ± 0.047
CatBoost	0.794 ± 0.075	0.862 ± 0.061	0.785 ± 0.083	0.768 ± 0.109	0.819 ± 0.102	0.149 ± 0.037
HistGBM	0.781 ± 0.062	0.847 ± 0.065	0.774 ± 0.070	0.764 ± 0.101	0.798 ± 0.090	0.168 ± 0.047

To formalize these observations, a non-parametric Friedman Test confirmed statistically significant differences among the models ($\chi^{2}=40.53,p<0.001$). Post-hoc Wilcoxon signed-rank tests, adjusted for multiple comparisons, indicated that CatBoost’s performance advantage, while numerically modest, was statistically significant compared to the other architectures (Table 3).

**Table 3:** Statistical Comparison of Model AUC Distributions.

Comparison	Median Difference	Statistic	p-value	Adj. p-value
Friedman Test (All Models)	–	40.53	< 0.001	–
CatBoost vs XGBoost	0.0156	942.0	< 0.001	< 0.001
CatBoost vs LightGBM	0.0195	799.0	< 0.001	< 0.001
CatBoost vs HistGBM	0.0156	851.5	< 0.001	< 0.001

### 3.2. Clinical utility and calibration

Fig 4 presents a comprehensive evaluation of model utility. The ROC curves (a) exhibit strong discriminatory capacity across all models, with consistent AUC values clustering around 0.90. Furthermore, the Precision-Recall curves (b) demonstrate high Average Precision (AP ≈0.93), indicating that the algorithms successfully maintain a high positive predictive value (few false positives) even at elevated sensitivity thresholds. The calibration curves (c) indicate that while the models generally capture the upward trend of risk, there is variation in probability estimates across specific bins, which is expected given the sample size constraints. The Decision Curve Analysis (DCA) (d) evaluates clinical applicability. Across a threshold probability range of approximately 0.10 to 0.80, all four algorithms maintained a net benefit above the default “Treat All” and “Treat None” baseline strategies. This suggests that applying these models could yield a positive clinical net benefit when used to stratify patients for further diagnostic testing. Note that, DCA evaluates whether model-assisted triage confers a net clinical benefit by weighing the value of correctly identifying gallstone cases for confirmatory imaging against the resource burden of unnecessary sonographic referrals. The observed net benefit across threshold probabilities of 0.10–0.80 indicates that using the model to guide ultrasound referrals captures more true cases while avoiding unneeded imaging compared to default strategies of referring all or no patients. This net benefit does *not* mean that definitive therapeutic or surgical decisions (such as cholecystectomy) should be initiated based on model predictions alone. Nor does it establish prospective real-world clinical efficacy or cost-effectiveness, which can only be confirmed through prospective clinical trials in routine practice.

**Fig 4:**
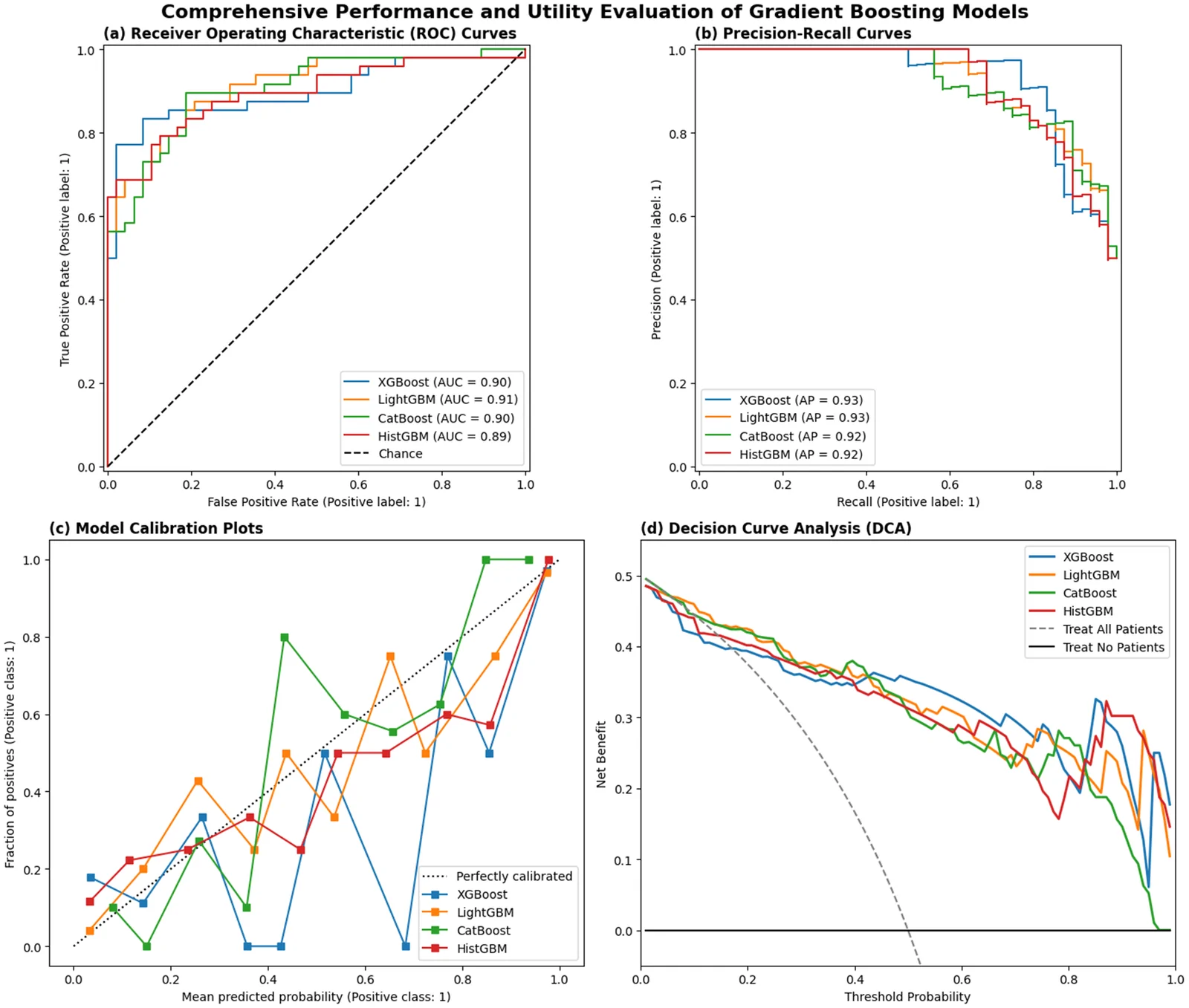
Comprehensive Performance Panel. (a) ROC Curves, (b) Precision-Recall Curves, (c) Model Calibration Plots, and (d) Decision Curve Analysis (DCA). The DCA demonstrates consistent net benefit for the models compared to baseline clinical strategies.

### 3.3. Model interpretability and feature importance

Global SHAP values were extracted to interpret feature influence (Fig 5). Across multiple boosting frameworks, CRP, Vitamin D, and BM consistently appeared as high-impact variables. High values of CRP were generally associated with increased gallstone risk, while lower Vitamin D levels contributed to the positive class prediction.

**Fig 5:**
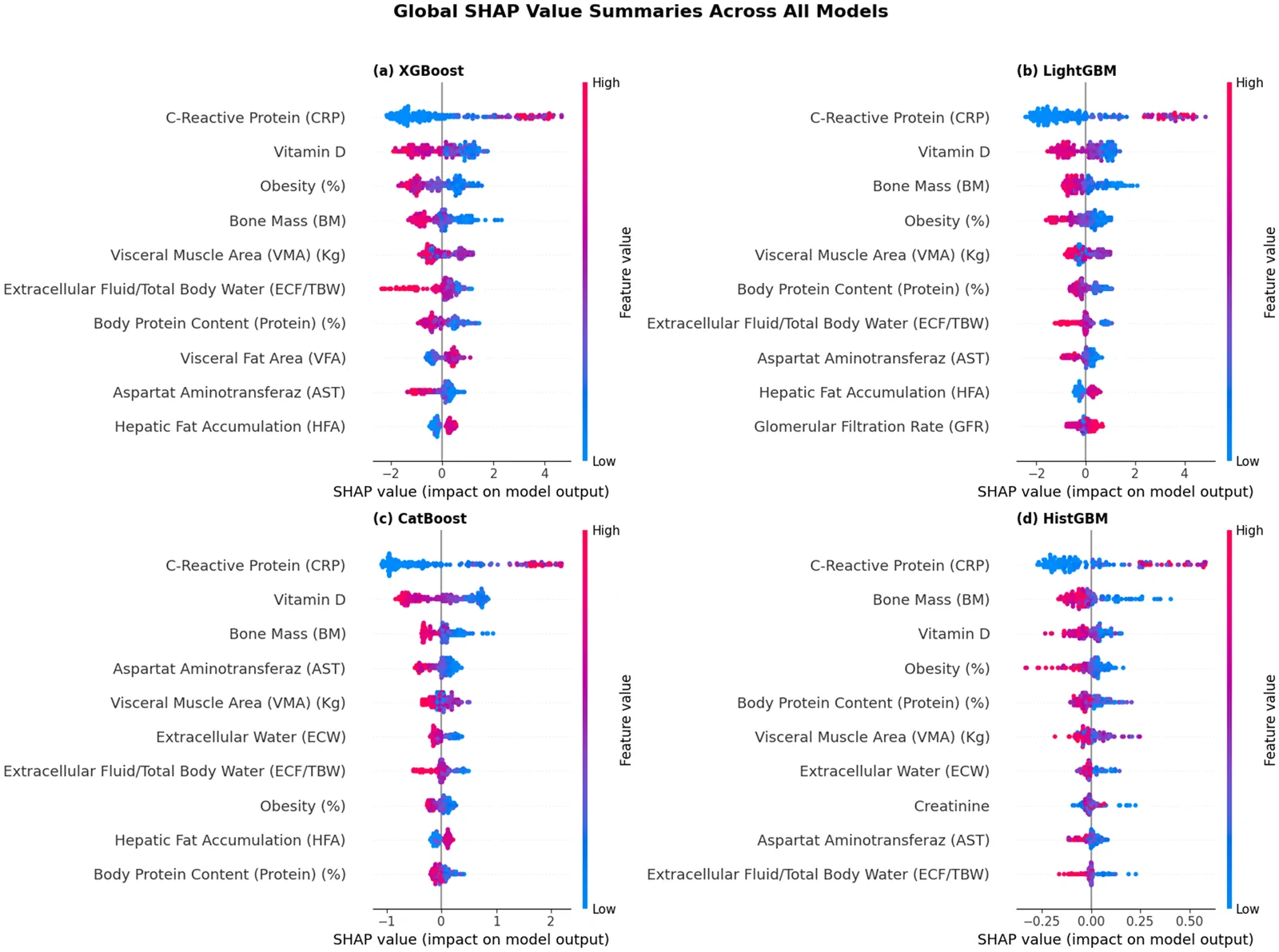
Global SHAP Value Summaries Across Models. Each point represents a patient; position on the x-axis indicates the impact on the model’s prediction, and color represents the original feature value.

Further exploration via SHAP dependence plots (Fig 6) visualized non-linear risk associations. For instance, the VFA displayed an inflection point where risk contribution transitioned from negative to positive. Interaction effects were also noted; the plot for BMI showed distinct risk stratifications based on gender, indicating that equivalent BMI scores may carry different predictive weights depending on patient sex.

**Fig 6:**
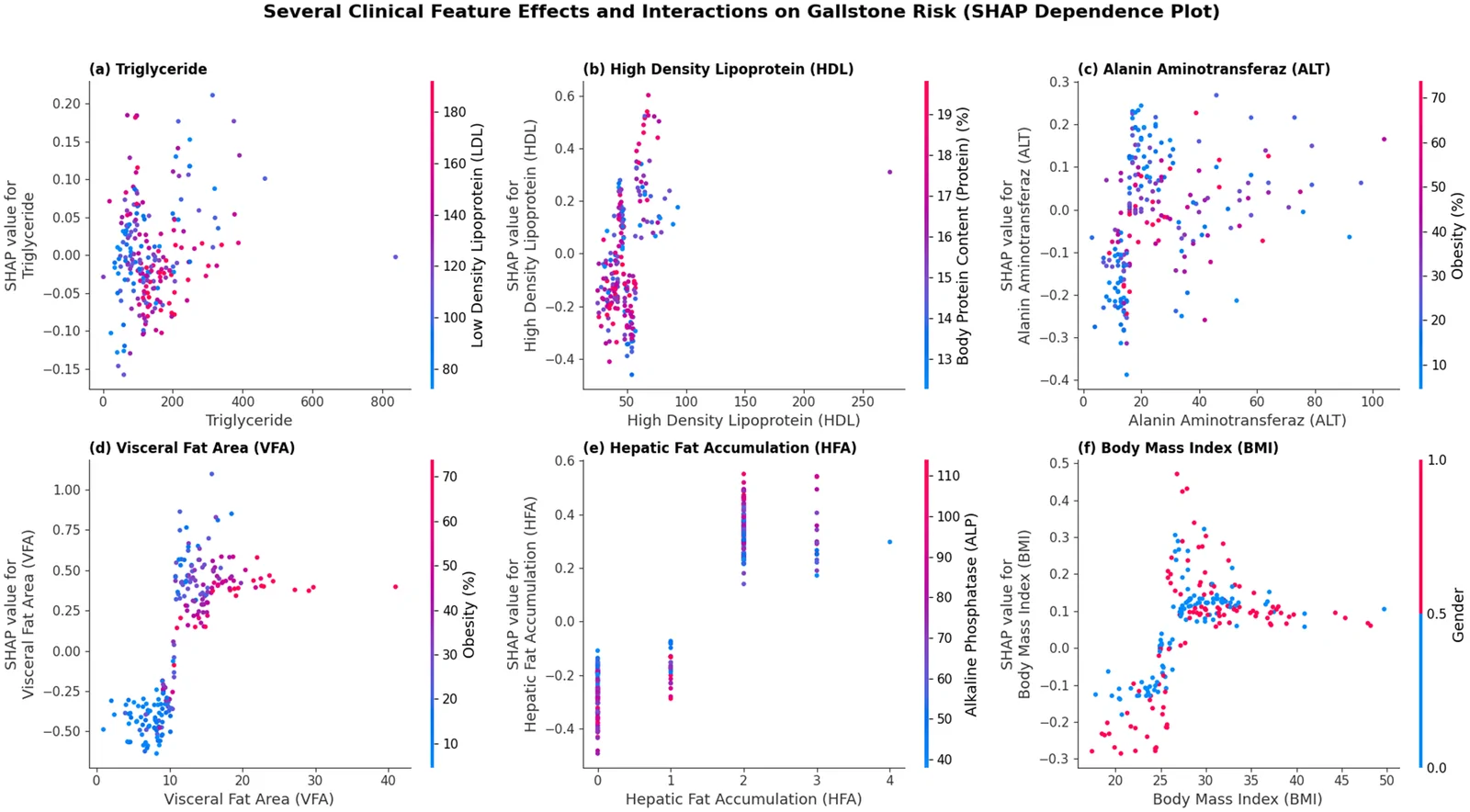
SHAP Dependence Plots illustrating non-linear feature effects and interactions. The y-axis shows the SHAP value (impact on prediction), while the right y-axis color bar denotes the automatically selected interacting feature.

### 3.4. Feature set ablation analysis

An ablation study was performed to assess the incremental and independent predictive value of the feature domains (Table 4). Models trained exclusively on basic demographics yielded an AUC of 0.476±0.097, indicating insufficient predictive capability. The standalone Bioimpedance (BIA) feature set demonstrated a strong independent signal (AUC = 0.797±0.079), approaching the performance of the traditional Laboratory set (AUC = 0.829±0.070). The integration of all feature sets yielded an overall AUC of 0.847±0.064, suggesting that BIA and serological markers capture complementary diagnostic information.

**Table 4:** Feature Group Ablation Study (evaluated via XGBoost).

Feature Set	AUC	F1 Score	Accuracy
Demographics only	0.476 ± 0.097	0.468 ± 0.102	0.485 ± 0.087
Labs only	0.829 ± 0.070	0.749 ± 0.081	0.755 ± 0.075
Bioimpedance only	0.797 ± 0.079	0.706 ± 0.086	0.722 ± 0.075
Combined (All Features)	0.847 ± 0.064	0.776 ± 0.075	0.782 ± 0.068

## 4. Discussion

### 4.1. Summary of findings

This study evaluated the predictive capacity of gradient boosting architectures for gallstone disease classification using a combination of point-of-care BIA and standard laboratory markers. Our findings suggest that machine learning algorithms, notably CatBoost, can achieve robust discriminatory performance (AUC up to 0.862) while offering potential clinical value. Furthermore, the ablation analysis highlighted that demographic profiling alone is insufficient for reliable classification. However, BIA parameters hold substantial independent predictive value, and their combination with laboratory features yields the most comprehensive risk assessment.

### 4.2. Clinical and pathophysiological insights

The application of explainable AI (SHAP) allowed for an examination of the features driving the algorithms’ predictions. Systemic inflammatory markers, notably CRP, alongside Vitamin D deficiency, were strongly associated with gallstone risk. This observation aligns with existing literature suggesting a role for inflammatory stasis and Vitamin D receptor activity in biliary tract motility [40,41].

Additionally, the models successfully leveraged specific adiposity metrics over generalized weight measures. The SHAP dependence plots for VFA and HFA illustrated non-linear thresholds where risk contribution notably increases. This supports the clinical understanding that localized, metabolically active central adiposity, which often contributes to insulin resistance and cholesterol supersaturation, is a more precise indicator of gallstone pathogenesis than standard BMI [42]. The algorithms were able to implicitly capture these established complex relationships, along with interactions between liver enzymes and obesity percentages.

### 4.3. Intended clinical context and potential utility

A critical consideration in evaluating the practical value of the proposed machine learning framework is defining its operational role relative to established diagnostic pathways. Transabdominal ultrasonography remains the accurate, and non-invasive first-line diagnostic standard for suspected gallstone disease. Consequently, the multi-domain gradient boosting models presented in this work are neither intended to supersede abdominal ultrasound nor suited for indiscriminate mass screening of asymptomatic individuals in the general population, an intervention that clinical practice guidelines uniformly discourage.

Instead, the clinical utility of these models is situated in opportunistic risk stratification and pre-imaging decision support across specific clinical workflows. The framework does not propose obtaining bioimpedance analysis and specialized serological panels solely to evaluate gallbladder status. Rather, it is designed to function as an automated, passive decision-support layer in clinical environments where metabolic panels, routine blood tests, and body composition assessments are already collected as part of standard care, such as bariatric clinics, metabolic syndrome management programs, and comprehensive health evaluations. In these environments, the model can synthesize co-occurring clinical parameters to flag individuals harboring elevated latent gallstone risk who might benefit from selective clinical evaluation. Additionally, in resource-constrained outpatient or primary care facilities facing prolonged sonography waitlists, patients frequently present with vague, non-specific dyspeptic or upper abdominal discomfort where cholelithiasis is among several competing differential diagnoses. In such ambiguous scenarios, the model could serve as an objective pre-test probability estimator, assisting clinicians in prioritizing patients with high predicted disease likelihood for expedited confirmatory imaging while supporting conservative management or alternative diagnostic evaluations in low-risk individuals. Patients presenting with acute, classic biliary colic should nonetheless bypass tabular predictive tools entirely and proceed directly to urgent sonographic examination.

### 4.4. Strengths and limitations

A principal strength of this study lies in its multi-domain integration of point-of-care bioimpedance parameters with routine laboratory markers, evaluated across four gradient boosting architectures under a rigorous repeated stratified cross-validation scheme. Combining discriminatory metrics with calibration assessments, DCA, and SHAP-based global and interaction interpretability provides both technical rigor and pathophysiological transparency.

Nevertheless, several important limitations must be acknowledged. Foremost is the retrospective, case-control composition of the study cohort, which compared individuals with confirmed gallstone disease directly against healthy controls. As a consequence, our findings demonstrate retrospective disease classification rather than prospective longitudinal risk prediction or population-level screening efficacy. This balanced case-control configuration does not reflect the natural community prevalence of gallstone disease, nor does it account for confounding gastrointestinal pathologies, such as peptic ulcer disease, functional dyspepsia, or non-alcoholic fatty liver disease, that are frequently encountered in routine diagnostic triage. Furthermore, because bioimpedance metrics and laboratory markers such as C-reactive protein and liver enzymes were collected cross-sectionally at the time of clinical evaluation, they reflect contemporaneous associations rather than definitive antecedent etiologies; elevated inflammatory indices, for instance, may represent subclinical reactive responses to established calculi. Additional methodological constraints include the single-center derivation of the cohort from an outpatient clinic and the observed bin-to-bin variance in empirical calibration curves. While the dataset proved adequate for tree-based ensemble learning, these calibration fluctuations suggest that post-hoc recalibration techniques, such as Platt scaling or isotonic regression, will be necessary prior to any clinical translation. Prospective multi-center validation in demographically diverse, unselected clinical populations remains mandatory to verify the generalizability of the established decision thresholds and feature importances.

## 5. Conclusion

This study evaluated four gradient boosting algorithms, XGBoost, LightGBM, CatBoost, and HistGBM, for the discrimination of gallstone disease using an integrative profile of demographic, bioimpedance, and laboratory features. Among the evaluated architectures, CatBoost demonstrated the strongest discriminatory capacity (AUC=0.862±0.061, accuracy=0.794±0.075) and lowest Brier score (0.149±0.037), showing statistically significant superiority over competing models (*p* < 0.001). Feature ablation revealed that bioimpedance parameters alone conferred substantial discriminative signal (AUC=0.797±0.079), approaching that of serological markers (AUC=0.829±0.070), whereas demographic variables alone were insufficient (AUC=0.476±0.097). SHAP interpretability analyses identified C-reactive protein, Vitamin D, and bone mass as primary drivers, alongside non-linear risk inflections for visceral fat area and body mass index. Crucially, the proposed models are not intended to replace abdominal ultrasonography as the definitive first-line diagnostic standard, nor do they support unselected screening of asymptomatic individuals. Instead, this work provides proof-of-concept evidence for opportunistic risk stratification in metabolic care environments where such data already exist, and for pre-imaging triage among patients presenting with ambiguous dyspeptic symptoms in resource-constrained settings. Because this study reflects retrospective classification within a curated case-control cohort, prospective longitudinal evaluations in broader clinical populations with diverse gastrointestinal differentials are essential to definitively establish real-world diagnostic yield and clinical utility.
